# Resourceful Utilization of Cow Hair in the Preparation of Iron Tailing-Based Foam Concrete

**DOI:** 10.3390/ma15165739

**Published:** 2022-08-19

**Authors:** Leipeng Liu, Junjie Yang, Yinfei She, Shenghua Lv, Zheng Yang, Jia Zhang

**Affiliations:** 1 College of Bioresources Chemical and Materials Engineering, Shaanxi University of Science and Technology, Xi’an 710021, China; 2 College of Chemistry and Materials Science, Weinan Normal University, Weinan 714099, China

**Keywords:** cow hair, foam concrete, mechanical performance, stomatal structure, chemical foaming

## Abstract

Cow hair, a by-product of tannery waste, is usually stockpiled on a large scale as waste, which leads to serious environmental impacts. In this paper, cow hair was used as a reinforcement fiber to improve the mechanical strength of iron tailing-based foam concrete. The effects of the amount of cow hair fiber on the apparent density, compressive strength, and flexural strength of foam concrete were investigated by a series of characterization methods. Meanwhile, Image-Pro Plus software was used to analyze the porosity, average pore size, roundness, and other parameters of the specimens with different amounts of cow hair fiber. Results revealed that a proper amount of cow hair fiber can form a stable three-dimensional network structure inside the foam concrete and promote a uniform distribution and size of the pore structure inside the test piece. This could effectively improve the compressive strength, flexural strength, and crack resistance of the foam concrete, and when the fiber content was 0.2 wt%, the foam concrete exhibited the best mechanical properties, with a compressive strength of 11.19 MPa and a flexural strength of 3.58 MPa. The present work was in agreement with the strategic objective of resource recycling and solid waste utilization, which was conducive to the development of the circular and green economy.

## 1. Introduction

Foamed concrete (FC) is a cement-based composite material with a low density (400 kg/m^3^~1800 kg/m^3^) and low thermal conductivity, and it is widely used in the non-load-bearing field in the building industry. However, the structural characteristics of light weight and porosity have also led to the generally low strength and toughness of foamed concrete [1], which has limited its wider application. The incorporation of fibers as reinforcement into foamed concrete has been proved to be an efficient method of reinforcing the mechanical properties of FC, considering that the randomly oriented fibers can bridge cracks from extension and deliver a greater load-carrying capacity of FC.

Past studies have shown that adding the appropriate amount of fiber during the preparation of foam concrete can effectively reduce the average pore size [2,3,4,5,6] and the shrinkage load of the material, leading to an increase in bending strength [7,8]. In general, the addition of fibers will lead to the transition from brittle failure to plastic failure of foam concrete; when the material cracks, the bridging effect between the fiber and the concrete matrix makes the material have a higher bearing capacity and plastic deformation capacity. At present, the more widely used concrete industry mainly includes inorganic fibers such as basalt fiber [9], steel fiber [10], glass fiber [11,12], and organic fibers of polypropylene fiber [13,14], and it is worth noting that some natural fibers can also reinforce foam concrete [15]. Asim et al. [16] mixed coconut, jute, sugarcane, sisal, and other natural fibers to prepare lightweight concrete composites, and they found that adding 2.5% of the total blend of coconut fiber and jute fiber could effectively improve the compressive strength and thermal insulation performance of FC. Pinto et al. [17] explored the possibility of using corncobs to improve the thermal insulation performance of lightweight building materials. La et al. [18] added an “ecological sandwich” prepared by mixing granular cork, flax fiber, and bio-based epoxy resin into the concrete system and successfully prepared a heat-insulating lightweight wall material with high thermal resistance and low thermal conductivity. Sa et al. [19] found that adding hemp fiber to concrete could effectively improve its compressive strength and flexural strength. Meanwhile, hemp fiber also makes a certain contribution to the improvement in concrete durability. Ali et al. [20] explored the influence of the length and content of coconut fiber on the mechanical properties of concrete. The results showed that when the fiber length was 5 cm and the fiber content was 5%, the effect of improving the mechanical properties of concrete was optimal. Osman Gencel [21] also used hemp fibers (HFs) to reinforce foam concrete, and its thermal conductivity, drying shrinkage, porosity, water absorption, and unit weight properties were studied. Many kinds of natural fiber have been used as reinforcement to improve the properties of foamed concrete, but there are almost no reports on the use of cow hair, a by-product of the leather industry, to reinforce FC.

The tanning industry is a traditional industry with a history of thousands of years. Cowhide is one of the most important types of leather, and cow hair is the main by-product of the tanning industry. Cow hair accounts for about 6% of the total weight of a cow, which means that a ton of salt wet cowhide will produce about 85 kg of cow hair [22]. The main material component of cow hair is keratin, and it also contains a small number of lipids and minerals. During the keratinization process of cow hair, the disulfide bridge between adjacent cysteine residues strengthens cortical keratin, which makes it difficult for cow hair to degrade naturally [23]. At present, the main treatment method for cattle hair is natural stockpiling, but the piling of a large amount of cattle hair waste will cause environmental problems. Therefore, it is necessary to reuse the tanning waste cattle hair. MR et al. [24] used cattle hair waste to prepare protein concentrates and explored the effects of different fermentation processes on the protein properties of the concentrate. Song et al. [25] used cattle hair to prepare porous biochar because of the high carbon content of cattle hair. However, those methods of using cow hair were tedious and used little cow hair. Moreover, cow hair exhibits a high elastic modulus of 1.53~1.98 GPa; thus, it has the potential to be used as reinforced material.

On the other hand, this is a continuous investigation to obtain alternative concrete-making materials involving economic, environmental, and social aspects to achieve sustainable development. For this reason, wastes such as fly ash [26], silica fume [27], waste marble dust [28,29], and ground blast furnace slag [30,31] are reused as cement substitutes and aggregates used in concrete. Inclusion of the wastes improves concrete performance, provides an environmental advantage, and reduces CO_2_ emissions [32,33].

To develop a new method that can apply tannery waste cow hair on a large scale, this paper tries to use cow hair as a fiber reinforcement material to prepare foam concrete, with iron tailings as the fine aggregate and P.O42.5 ordinary Portland cement as a cementitious material, and then explores the influence of the content of cow hair fiber on the density and mechanical properties of the foam concrete. Meanwhile, with the help of the software Image-Pro Plus, the influence of the cow hair fiber on the porosity, average pore size, and roundness of the pores in the foam concrete is comprehensively analyzed. Utilizing cow hair as a reinforcement fiber in foam concrete is remarkably important in terms of environmental preservation and sustainable development.

## 2. Materials and Methods

### 2.1. Materials

**Figure 1:**
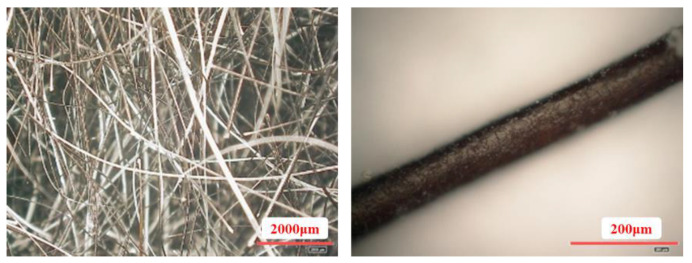
Photos of CHF under different magnifications.

Foamed concrete is mainly prepared by mixing cementitious materials, aggregates, and admixtures (foaming agent, foam stabilizer, coagulant, water reducing agent). In previous studies, the introduction of iron tailings with relatively low pozzolan activity and a certain hardness as fine aggregates into foam concrete systems can effectively improve the mechanical properties of the materials [34,35,36]. Therefore, the foamed concrete prepared in this experiment used iron tailings from an iron ore dressing plant in Pinquan, Hebei as the fine aggregate and P.O42.5 ordinary Portland cement produced by Shaanxi Shengwei Building Materials Co., Ltd. (Xianyang, China) as the cementing material. The hydrogen peroxide produced by Sinopharm Chemical Co., Ltd. (Shanghai, China) was selected as the foaming agent; the carboxypropyl methylcellulose (HPMC) produced by Henan Zhongyi Xinghui Chemical Reagent Factory(Zhongyi, China) was used as the foam stabilizer; sodium meta aluminate produced by Tianjin Bailunsi Biotechnology Co., Ltd. (Tianjin, China) was selected as the coagulant; and the water-reducing agent selected was the naphthalene-based water-reducing agent from Tianjin Fuchen Chemical Reagent Factory (Tianjin, China). Finally, as shown in Figure 1, the cattle hair fiber (termed CHF) used in this experiment was obtained from waste buffalo hair produced in the tanning industry after pretreatment.

### 2.2. Mixtures and Methods

**Figure 2:**
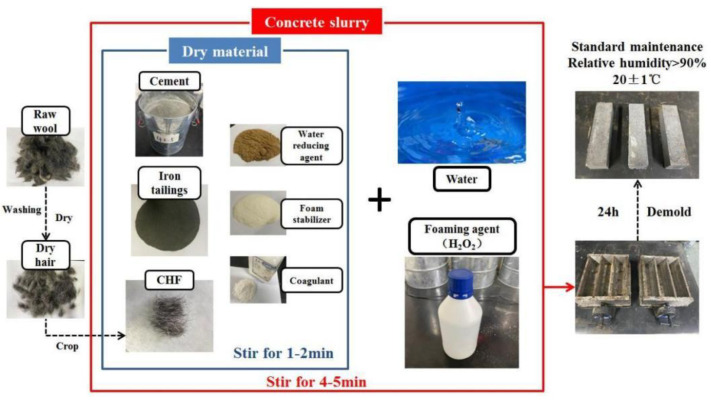
Schematic diagram of the preparation process.

The preparation process of the cow hair fiber foamed concrete involved in this experiment can be divided into three steps (Figure 2). The specific operations are as follows. First, wash off the surface grease of the waste cow hair and dry it; cut the washed raw wool into short fibers with a length of 15 ± 2 mm. Then, mix the cement and the iron tailings in proportion, add the external powder additives (HPMC, sodium meta aluminate, and naphthalene-based water reducer) and the treated CHF, and mix them evenly. Finally, add H_2_O_2_ and water to the above mixture and stir quickly for 5 min; then, pour the slurry into a 40 mm × 40 mm × 160 mm mold. After standard curing for 24 h, demold the sample and place it in the curing box for standard curing (relative humidity > 90%, 20 ± 1 °C). In this study, the influence of the amount of CHF on the performance of foam concrete was explored. As shown in Table 1, the prepared foam concrete specimens of each group were prepared with CHF contents of 0 wt%, 0.05 wt%, 0.10 wt%, 0.15 wt%, 0.20 wt%, 0.30 wt%, and 0.40 wt% and are named N_0_, N_0.05_, N_0.1_, N_0.15_, N_0.2_, N_0.3_, and N_0.4_, respectively.

### 2.3. Characterization

#### 2.3.1. Apparent Density

The density of the specimen was tested according to the standard JC/T 1062-2007. The apparent density of the sample needs to be tested after placing sample with a curing age of 28 days in an oven at 110 ± 5 °C to a constant weight. The apparent density *ρ* of the sample was calculated using the formula *ρ = M/V*, where *M* represents the mass of the sample after drying, and *V* represents the sample volume.

#### 2.3.2. Compressive Strength

The compressive strength of the specimen was tested according to the standard GB/T50107. The compressive strength of the sample was tested using an electro-hydraulic pressure testing machine. The compressive strength *R* of the sample can be determined according to the formula *R = F/A*, where *F* represents the breaking load of the sample, and *A* represents the compressed area of the sample.

#### 2.3.3. Flexural Strength

The flexural strength of the specimen was tested according to the standard GB/T50081-2002. A universal electric servo testing machine was used to measure the flexural strength of the test piece. The flexural strength *f* of the test piece can be determined according to the formula *f* = *3 FL*/*(2bh^2^)*, where *F* is the maximum load value, *L* is the span, *b* is the section width of the test piece, and *h* is the height of the section of the test piece.

#### 2.3.4. Stomatal Structure

In this study, a high-definition camera was used to photograph the pore morphology of each specimen under a fixed position, a fixed focal length, and a fixed exposure mode. PS software was used to capture photos of each group of specimens into 5 cm × 5 cm images. Then, each group of pictures was imported into the Image-Pro Plus software for pre-processing, such as background removal, color correction, calibration ruling, color selection, and binarization. Finally, the analysis and calculation functions of the software were used to calculate the porosity, average pore diameter, and roundness of each group of specimens.

#### 2.3.5. Porosity

To calculate the porosity of the sample, it is necessary to first test its true density. The sample, cured for 28 days and dried to a constant weight, was ground and tested using the T0352-2000 mineral powder density test method (Li’s bottle method) to obtain the true density of the sample as *ρ*_0_. The porosity *P* was calculated using the formula *P = (1 − ρ/ρ*_0_*) × 100%*, where *ρ* represents the block density of the sample.

For the test of apparent density, compressive strength, flexural strength, and porosity of the foam cement, each sample was tested five times, and the average value was taken.

## 3. Results and Discussion

### 3.1. Raw Material Analysis

#### 3.1.1. Cow Hair Fiber (CHF)

The uniformly dispersed fibers can form a chaotically distributed three-dimensional network structure inside the foam concrete system, effectively arresting growth and widening of cracks in the material and improving the material’s mechanical properties. The fiber’s aspect ratio and tensile strength are the keys to its function in foam concrete. The specific performance indices of the CHF used in this experiment are shown in Table 2.

#### 3.1.2. Iron Tailings

**Figure 3:**
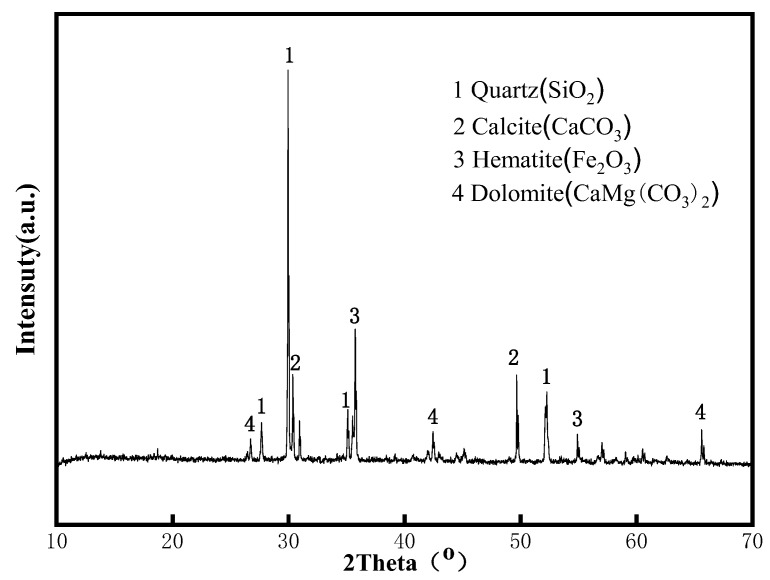
The ancestry of the iron tailings.

The particle size of the iron tailing sand used in this experiment was mainly distributed in the range of 70–110 μm; the ignition loss was about 2.13%, and its chemical composition is shown in Table 3. Meanwhile, using X-ray diffraction (XRD) to analyze the phase composition of the iron tailings, it was found that the iron tailings selected for this experiment were mainly composed of quartz (SiO_2_), calcite (CaCO_3_), hematite (Fe_2_O_3_), and dolomite (CaMg(CO_3_)_2_). The specific XRD spectrum is shown in Figure 3.

#### 3.1.3. Stomatal Structure

Foam concrete is a cement-based composite material with a large number of pores on the inside. The pore structure inside foam concrete is one of the crucial parameters of FC, for it is closely related to the strength and porosity of the foam concrete [37,38,39]. The pore structure of FC was usually analyzed through the porosity, average pore size, pore size distribution, and circularity value of the material.

(1) Overview of Pore Structure

**Figure 4:**
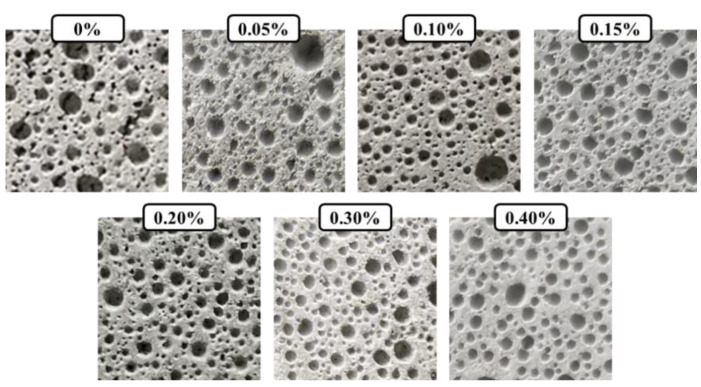
The pore structure of CHF foam fibers captured by a high-definition camera.

**Figure 5:**
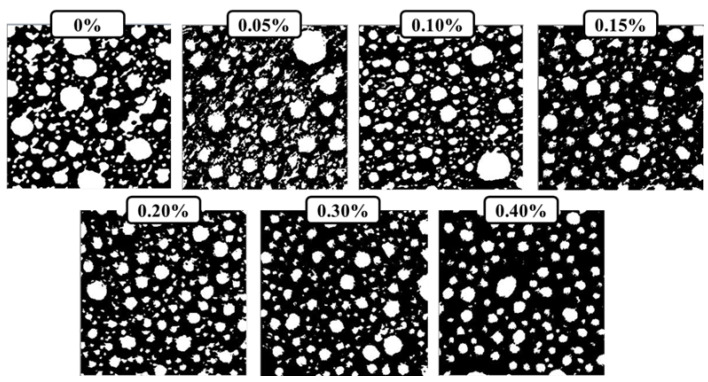
The pore structure of CHF foam fiber after binarization using IPP software.

A high-definition camera was employed to observe the pore structure of the material. Before shooting, 180-mesh, 400-mesh, and 1200-mesh sandpapers were used to polish the sections of the specimens, and the parts were smooth and visible to the naked eye. After testing the CHF, a soft brush was used to brush off the dust blocked in the air holes as much as possible to avoid affecting the test results. After the shooting, PS software was employed to cut each picture into a size of 5 cm × 5 cm, as shown in Figure 4. Then, IPP software was used to binarize each group of pictures (Figure 5). After selecting the pores of each group of pictures, the software was used to calculate the major characteristic parameters of the pore structure.

**Figure 6:**
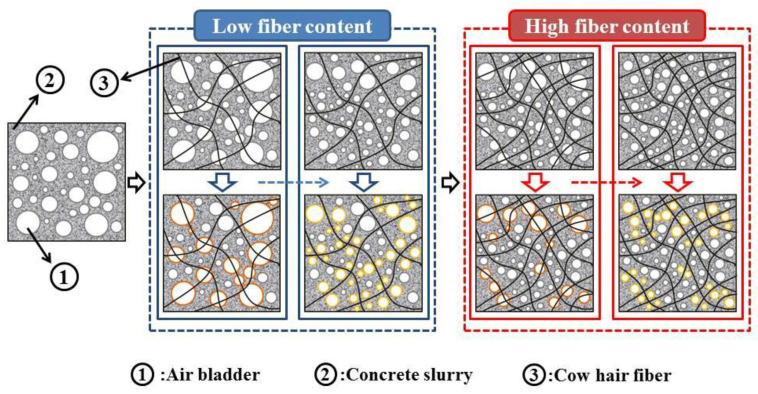
The mechanism of CHF affects the pore structure of foamed concrete.

As shown in Figure 5, the addition of CHF affected the pore structure of the foam concrete. When there was no CHF added, the large-diameter air bubbles in the foam concrete accounted for a large proportion, and there was also a large number of small-sized interconnecting air holes. The pore size deviation of the pores was large. With the incorporation of CHF, the original interconnected pores gradually disappeared, replaced by a large number of independent small-sized pores. At the same time, the size of the original larger pores was also reduced. With the increase in CHF content, the pore diameter of the small-sized pores gradually increased; the pore diameter of the large-sized pores gradually decreased; and the pore diameter deviation of the overall pores gradually decreased. Therefore, the addition of an appropriate amount of CHF can effectively promote the formation of a uniformly dispersed and uniform pore structure in the foam concrete. We think that the mechanism of this phenomenon may be that the CHF can form a 3D network in the foam concrete, and, during the chemical foaming process of the concrete slurry, bubbles will inevitably aggregate, which makes the size of the bubbles increase inside the slurry to varying degrees, as shown in Figure 6. The interwoven network structure of CHF can divide some relatively large bubbles into two or more relatively small bubbles, and, with the increase in fiber content, this network structure also becomes denser. Under the action of continuously dividing and rearranging, the aggregation and enlargement of the bubbles becomes restrained effectively. After the slurry is solidified and formed, a fine and uniform pore structure is generated inside the foamed concrete.

(2) Porosity

**Figure 7:**
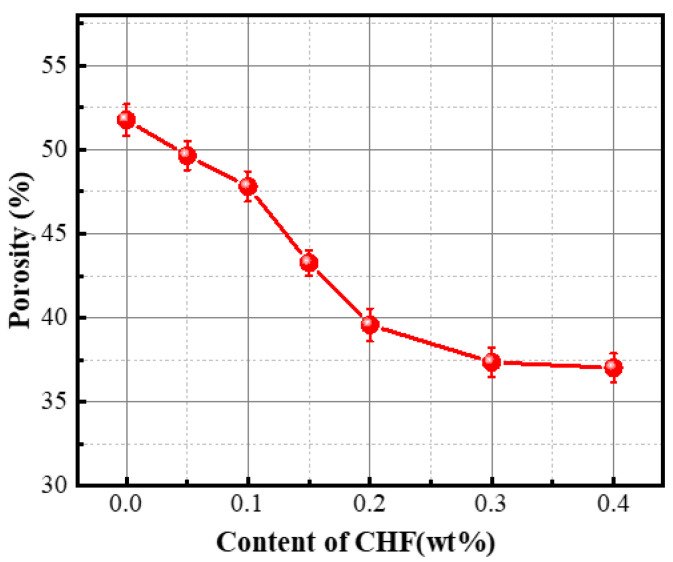
Influence of the content of CHF on the porosity of the material.

The effect of CHF content on the porosity of foamed concrete is shown in Figure 7. With the gradual increase in CHF content, the porosity of foamed concrete gradually decreased, but when the content of CHF was up to 0.3% and above, the porosity of FC remained approximately constant. In the pore structure overview part, it was found that the addition of CHF made the air pores inside the foam concrete smaller and the interconnected air pores disappear, thereby effectively promoting the uniformity of air pore size in the foam concrete. Therefore, the addition of CHF makes the porosity of foam concrete decrease sharply up to 0.2%, and, with the further increase in CHF content, the decreasing trend of porosity tends to be gentle.

(3) Average pore size

**Figure 8:**
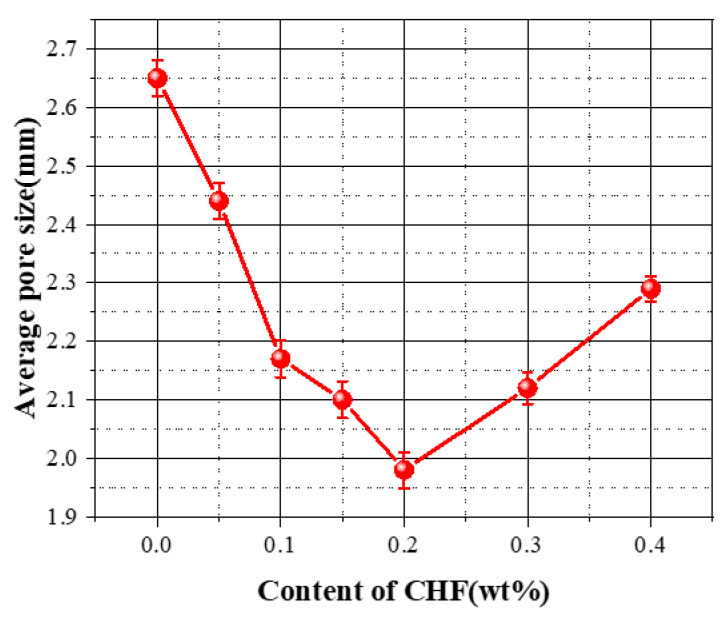
Influence of the content of CHF on the average pore size of the material.

**Figure 9:**
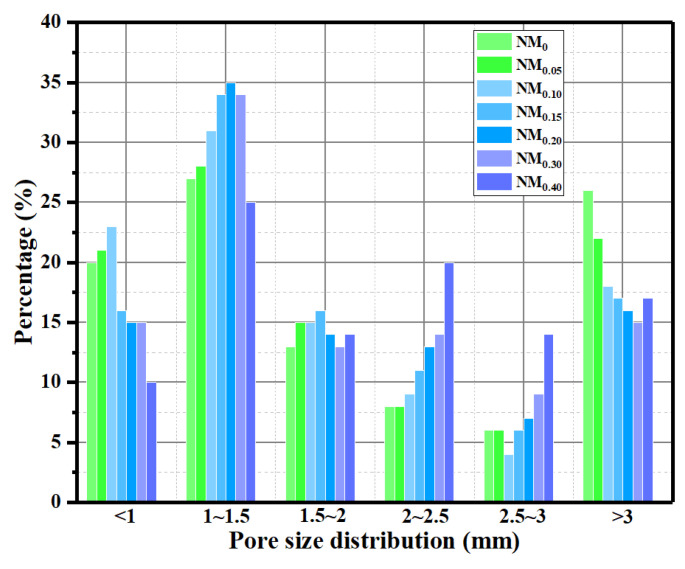
Influence of the content of CHF on the pore size distribution of the material.

**Figure 10:**
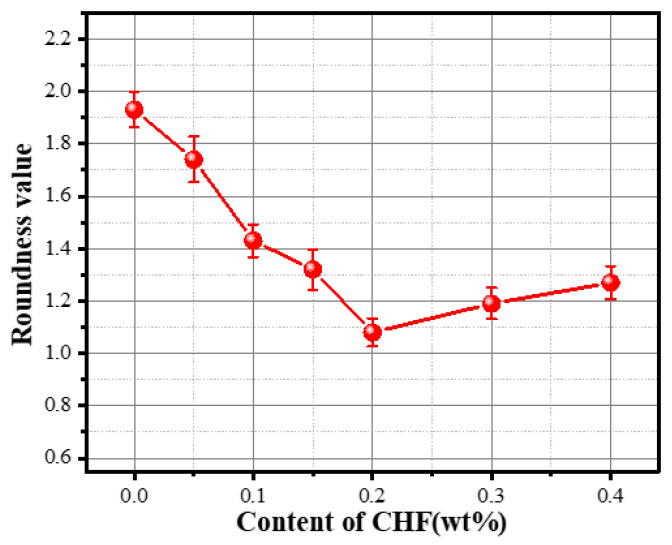
Influence of the content of CHF on the roundness value of the material.

The effect of CHF content on the average pore size of foam concrete is shown in Figure 8. With the increase in CHF content (from 0 wt% to 0.2 wt%), the average pore size of FC showed a downward trend. However, with further increases in the content of bovine hair from 0.2 wt% to 0.4 wt%, the average pore size of the material increased. The reason for this phenomenon needs to be comprehensively analyzed in combination with the pore size distribution of each group of specimens; as shown in Figure 9, when the CHF content ranged from 0.05 wt% to 0.2 wt%, the pore diameters inside the specimen were mainly distributed in the smaller size range of <1 mm, 1~1.5 mm, and >3 mm. The larger size range of pore diameters proves that the lower fiber content had limited restraint on the pore size of the material. The distribution to 1.5~3 mm proves that the appropriate fiber content can effectively eliminate the small-sized interconnected pores inside the material and also restrain the generation of large-sized bubbles. When the CHF content was further increased (0.2~0.4 wt%), the pores inside the specimen showed higher pore sizes in the three pore size ranges of 1.5~2 mm, 2~2.5 mm, and 2.5~3 mm. According to the comprehensive analysis in Figure 2, Figure 3, Figure 4, Figure 5, Figure 6, Figure 7, Figure 8, Figure 9 and Figure 10, when the CHF content increased from 0.3 wt% to 0.4 wt%, the porosity of the specimen changed very little. The high proportion of CHF was the main reason for the increase in the average pore size in the FC specimens when the CHF content was 0.2~0.4 wt%.

(4) Roundness value

The roundness value, the degree of the pores close to a circle, is a characteristic parameter used to characterize the geometric shape of the pores in foam concrete [40]. When the roundness value S is equal to 1, this means that the geometry of the pores is a regular sphere. The closer the average roundness value of all pores is to 1, the more regular in shape the pores inside the FC are and the better the mechanical properties.

The effect of CHF content on the roundness value of the foam concrete is plotted in Figure 10. With the increase in CHF content, the roundness value of foam concrete cells first decreased and then increased; when the content of CHF was 0.2 wt%, the roundness value of the specimen was closest to 1. In the absence of CHF, the generation of air bubbles in the foaming process of the concrete slurry is not restricted, resulting in a large number of connecting pores in the final material and poor regularity of large-sized air bubbles. With the gradual incorporation of CHF, the interconnected pores in the specimen gradually disappeared, and the large-sized bubbles gradually decreased and tended to be regular in shape under the constraint of the three-dimensional network structure of the fibers, which led to a gradual decrease in the roundness value of the material. The addition of excess fibers will cause a defoaming effect, which is also one of the reasons for the increase in the roundness value of the material.

### 3.2. Influence of the Content of CHF

**Figure 11:**
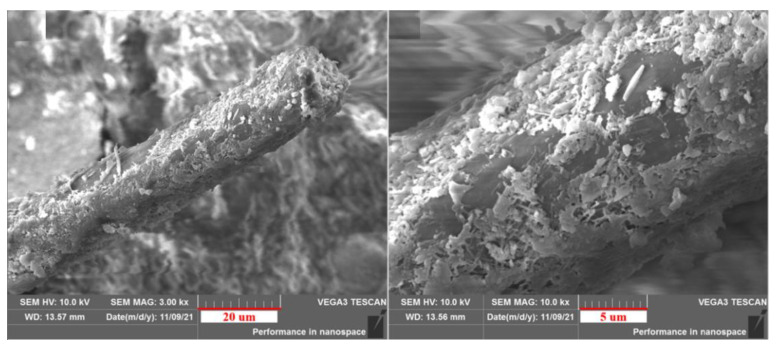
The micromorphology of cow hair fiber inside the material.

The microscopic morphology of the FC composite is shown in Figure 11. It was found that they were without obvious cracks between the cow hair and the concrete. Moreover, the needle-like hydration products were generated in the scaly layer of cow hair fiber and coated on its surface, which indicates that the rough surface of the cow hair fiber was closely connected with cement hydration products. This is also the main reason why cow hair fiber can improve the strength and toughness of materials.

#### 3.2.1. Apparent Density

**Figure 12:**
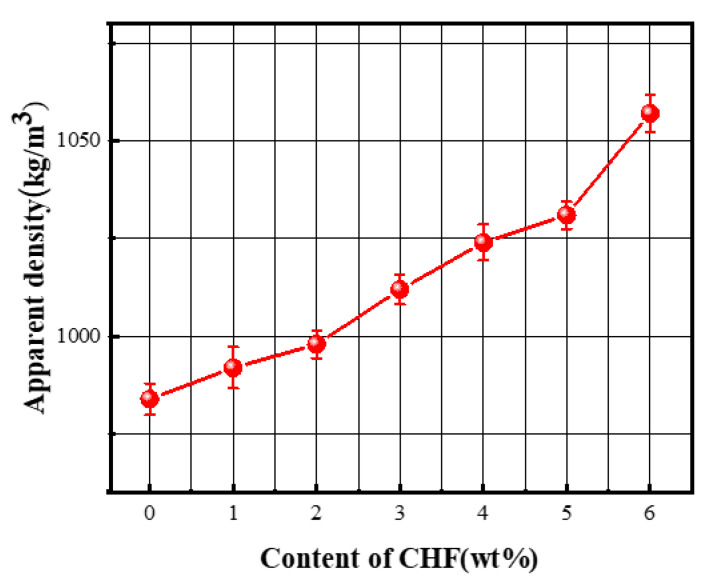
Influence of the content of CHF on the apparent density of the material.

Figure 12 shows the effect of CHF content on the apparent density of foamed concrete. With the gradual increase in CHF content, the apparent density of the specimen showed a slight upward trend (from 984 kg/m^3^ to 1057 kg/m^3^), and this result was consistent with the change in porosity mentioned above. This phenomenon was also caused by the CHF three-dimensional network structure inside the concrete slurry, which limited the generation of large-sized pores. Additionally, adding CHF will affect the fluidity of the slurry, and the decreased fluidity will lead to the defoaming phenomenon inside the foamed concrete.

#### 3.2.2. Compressive Strength

**Figure 13:**
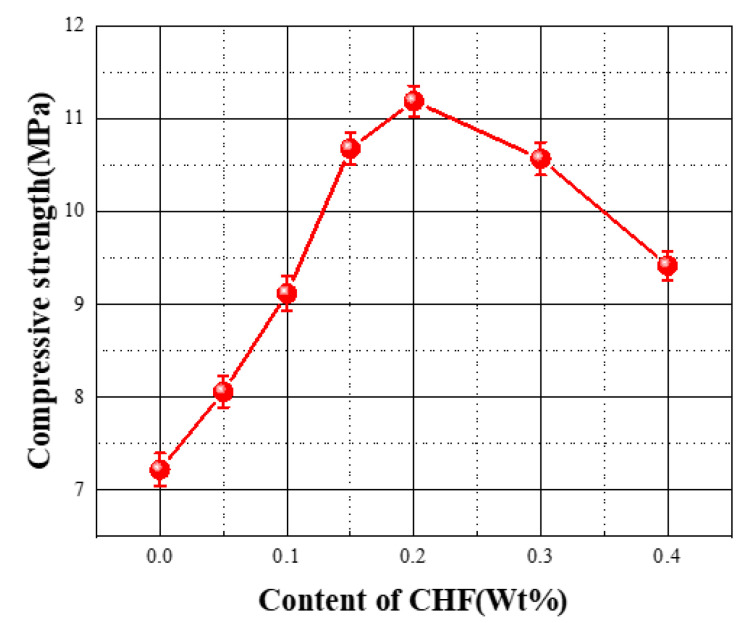
Influence of the content of CHF on the compressive strength of the material.

The compressive strength of the foam concrete is plotted in Figure 13. The compressive strength of FC without CHF was only 7.22 MPa. The compressive strength of the specimen increased gradually with the increasing CHF content and reached a maximum value of 11.19 MPa when the CHF content was 0.2 wt%, a cumulative increase of 68.37%. Subsequently, when the CHF content further increased from 0.2 wt% to 0.4 wt%, the compressive strength of the specimen decreased from 11.19 MPa to 9.42 MPa, but this value was still higher than that of the blank specimens. This means that CHF exhibits an enhancing effect on the compressive strength of FC, for the cow hair fiber reinforcement enables a distributed growth of microcracks in the specimen before the development of macrocracks [41]. When the CHF content ranged from 0.05 wt% to 0.2 wt%, the three-dimensional network structure formed by the fibers effectively promoted the gradual formation of a pore structure with uniform distribution and size inside the FC, and the stable pore wall structure endowed the FC with good mechanical properties. Thus, the compressive strength of the specimens gradually increased with the increase in CHF, but when the CHF content increased from 0.2 wt% to 0.4 wt%, the dispersibility of the CHF in the concrete slurry continued to decrease. A large amount of fiber incorporated will also affect the crystallinity of the hydration product, resulting in a decrease in the strength of the specimen.

#### 3.2.3. Flexural Strength

**Figure 14:**
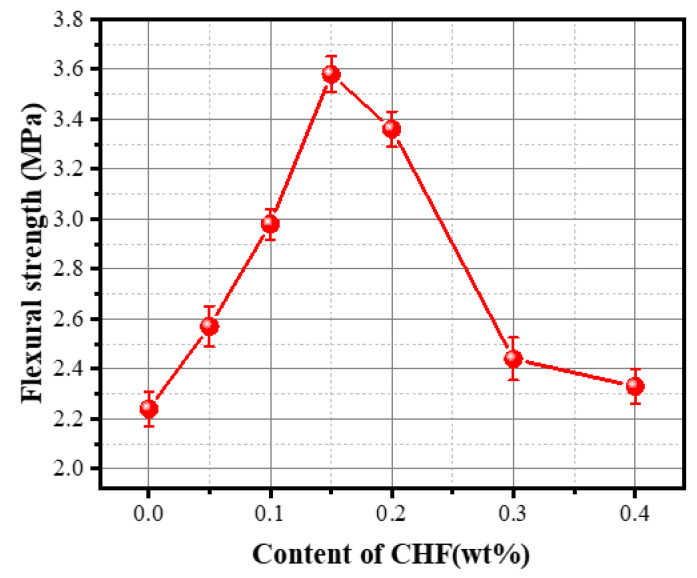
Influence of the content of CHF on the flexural strength of the material.

Figure 14 shows the effect of CHF content on the flexural strength of the foam concrete. With the gradual increase in CHF content, the 28-day flexural strength of the specimen showed a trend of first increasing and then decreasing. The flexural strength of the FC without CHF was 2.24 MPa. With the addition of CHF, the flexural strength of the specimen increased gradually and reached a maximum value of 3.58 MPa when the CHF content was 0.15 wt%, a cumulative increase of 59.82%. Subsequently, when the CHF content increased from 0.15 wt% to 0.4 wt%, the flexural strength of the specimen decreased from 3.58 MPa to 2.33 MPa. The changing trend of flexural strength of the FC is similar to that of the compressive strength of the specimen; the difference is that the maximum flexural strength of the specimen appeared when the content of cattle wool was 0.15 wt%, and a similar result has been reported elsewhere [41]. The reasons and laws for the enhancement of the flexural strength of the FC specimen are the same as for the effect of fibers on the compressive strength of the material. An appropriate amount of fiber can produce a stable bridging effect with the concrete matrix, and the fiber bridging action helps in transferring the tensile stress across the crack, thereby increasing the flexural strength of the fiber-reinforced FC [42].

However, with the increase in CHF content from 0.15 wt% to 0.4 wt%, under the condition of constant cement content, the amount of cementitious material used for the fiber–concrete bonding interface gradually increased with the increase in CHF content, resulting in defects in the pore wall of the material; the influence of excessively added fibers on the crystallinity of cement hydration products is also one of the reasons for the reduction in the flexural strength of the specimens.

**Figure 15:**
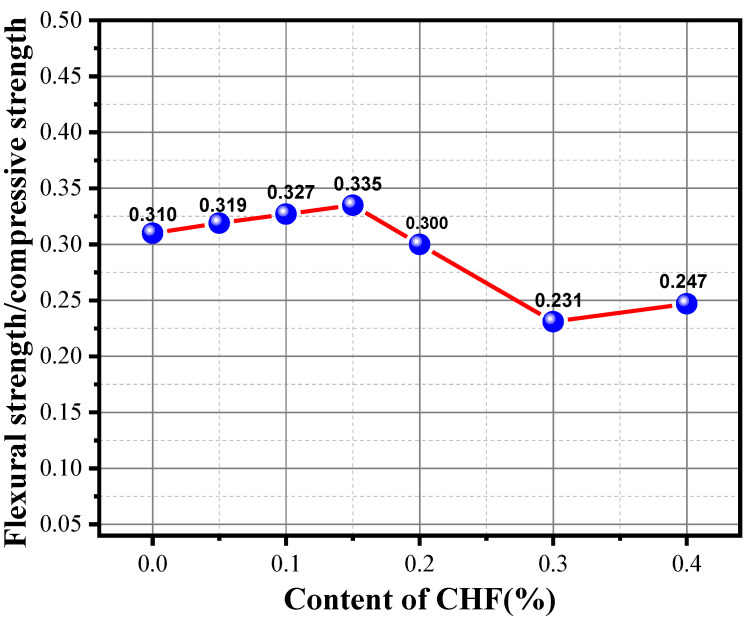
Influence of the content of CHF on the F/C of the material.

The ratio of the flexural strength and compressive strength (F/C) is an important parameter of concrete to explore its crack resistance. A large F/C value means that the material exhibits strong flexural resistance. As shown in Figure 15, it can be seen from the figure that, with the continuous incorporation of CHF, the F/C of the specimen showed a trend of first increasing, then decreasing, and then increasing. It is worth noting that the F/C of the FC specimen reached a maximum value of 0.335 when the content of cattle hair was 0.2 wt%, which indicates that the appropriate CHF content can effectively improve the crack resistance of the material, which is closely related to the compressive strength and flexural strength of the specimen. The results of the analysis of the flexural strength were consistent, and the excessive incorporation of CHF impaired the mechanical properties of the FC specimen. Therefore, the comprehensive analysis shows that the specimen had the best mechanical properties when the CHF content was 0.2 wt%.

**Figure 16:**
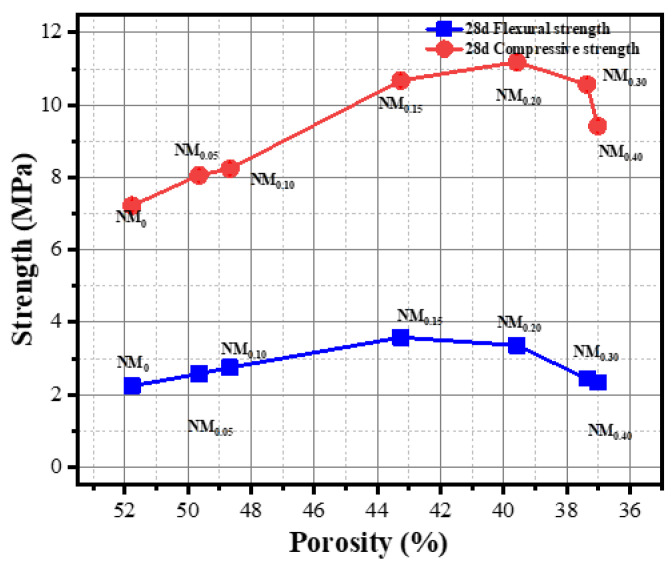
Corresponding relationship between porosity and strength of CHF foam concrete.

The change in the porosity of foamed concrete will influence its strength. Figure 16 shows the corresponding relationship between the porosity of the foamed concrete and its 28-day compressive strength and flexural strength.

The relationship between the density and the compressive strength and flexural strength of the cow hair fiber-reinforced foam concretes was linear when the addition of CHF was between 0 and 0.2 wt%, and this result had good agreement with the findings given elsewhere [43]. This shows that a reduction in porosity will thicken the pore wall structure inside the foam concrete, thereby effectively improving the strength of the material. However, when the content of CHF was up to 0.3% and above, with a further reduction in porosity, the strength of the material showed a slight downward trend. It may be concluded that the higher CHF content will cause problems, such as agglomeration and uneven dispersion, in the foam concrete and that the agglomerated CHF will lead to the cement matrix inside and around it having uneven dispersion, which will affect the hydration of the cement and cause internal defects in the specimen, thus affecting the strength development and behavior under stress.

## 4. Conclusions

In this paper, the CHF content was used as a single-factor variable, and the apparent density, compressive strength, flexural strength, and pore structure (porosity, average pore diameter, roundness value) of the foam concrete were used as experimental parameters to explore the effect of CHF content on foam concrete. Considering the impact on each performance, the specific conclusions are as follows.

1. Compared with the blank control group, all foamed concretes incorporating CHF showed higher compressive strength, flexural strength, and crack resistance, proving that CHF can improve the mechanical properties of foamed concrete. The compressive strength of the specimen reached a maximum value of 11.19 MPa when the CHF content was 0.2 wt%; the flexural strength reached a maximum value of 3.58 MPa when the CHF content was 0.15 wt%; and the fold-to-compression ratio reached a maximum value of 0.335 when the CHF content was 0.2 wt%.
2. With the gradual increase in CHF content, the porosity of the foamed concrete continued to decrease. When the CHF content was 0.2 wt%, the average pore diameter and pore roundness of the specimens decreased to minimum values of 1.92 mm and 1.08, respectively.
3. After a comprehensive analysis of the mechanical properties of foam concrete, it was found that, when the CHF content was 0.2 wt%, the foam concrete had the best mechanical properties; the compressive strength of the specimen was 11.19 MPa (an increase of 68.37%), the flexural strength was 3.36 MPa (increased by 50%), and the fold-to-compression ratio was 0.335 (increased by 8.06%). At the same time, after a comprehensive analysis of the characteristic parameters of the pore structure of the foamed concrete, it was found that the specimen had the most stable pore structure in the range of CHF contents ranging from 0.2 wt% to 0.3 wt%. Within this dosage range, the pores in the foam concrete were evenly distributed and uniform in size, and the stable pore wall structure also provided good mechanical properties of the specimen.

## Figures and Tables

**Table 1:** Mixing proportions.

ID	Cement/g	Iron Tailing/g	H_2_O_2_/g	CHF/g	Water/g
N_0_	450	150	11.25	0	225
N_0.05_	450	150	11.25	0.3	225
N_0.1_	450	150	11.25	0.6	225
N_0.15_	450	150	11.25	0.9	225
N_0.2_	450	150	11.25	1.2	225
N_0.3_	450	150	11.25	1.8	225
N_0.4_	450	150	11.25	2.4	225

**Table 2:** The performance of the CHF used in the test.

Diameter (μm)	Elastic Modulus (GPa)	Tensile Strength (MPa)	Elongation at Break (%)
85–115	1.53–1.98	97–121	19.23–26.47

**Table 3:** Chemical composition of iron tailings (IT).

IT	SiO_2_	Al_2_O_3_	Fe_2_O_3_	CaO	MgO	K_2_O	Na_2_O	Mn_2_O_3_	P_2_O_5_	TiO_2_
wt%	42.92	4.64	15.04	20.29	11.99	0.32	3.13	0.2417	0.4124	1.0275

## Data Availability

Not applicable.
